# Facile synthesis and defect optimization of 2D-layered MoS_2_ on TiO_2_ heterostructure for industrial effluent, wastewater treatments

**DOI:** 10.1038/s41598-020-78268-4

**Published:** 2020-12-10

**Authors:** Ramalingam Gopal, Maria Magdalane Chinnapan, Arjun Kumar Bojarajan, Naresh Kumar Rotte, Joice Sophia Ponraj, Ravi Ganesan, Ivanov Atanas, Manivannan Nadarajah, Rajesh Kumar Manavalan, Joao Gaspar

**Affiliations:** 1 Quantum Materials Research Lab (QMRL), Department of Nanoscience and Technology, Alagappa University, Karaikudi, Tamil Nadu 630003 India; 2 Department of Chemistry, St.Xavier College (Autonomus), Tirunelveli, Tamil Nadu 627002 India; 3 Centre for Advanced Materials, Integrated-Inter-Department of LiWET Communications, Aaivalayam - Dynamic Integrated Research Academy and Corporations (A-DIRAC), Coimbatore, 641046 India; 4 Department of Micro and Nanofabrication, INL–International Iberian Nanotechnology Laboratory, 4715-330 Braga, Portugal; 5 Department of Physics, Alagappa University, Karaikudi, Tamil Nadu 630003 India; 6 Department of Mechanical, Aerospace and Civil Engineering (MACE), Brunel University, Uxbridge, UK; 7 Department of Design, Brunel University, Uxbridge, UK; 8 Institute of Natural Science and Mathematics, Ural Federal University, Yekaterinburg, Russia 620002

**Keywords:** Materials science, Nanoscience and technology

## Abstract

Current research is paying much attention to heterojunction nanostructures. Owing to its versatile characteristics such as stimulating morphology, affluent surface-oxygen-vacancies and chemical compositions for enhanced generation of reactive oxygen species. Herein, we report the hydrothermally synthesized TiO_2_@MoS_2_ heterojunction nanostructure for the effective production of photoinduced charge carriers to enhance the photocatalytic capability. XRD analysis illustrated the crystalline size of CTAB capped TiO_2_, MoS_2_@TiO_2_ and L-Cysteine capped MoS_2_@TiO_2_ as 12.6, 11.7 and 10.2 nm, respectively. The bandgap of the samples analyzed by UV–Visible spectroscopy are 3.57, 3.66 and 3.94 eV. PL spectra of anatase phase titania shows the peaks present at and above 400 nm are ascribed to the defects in the crystalline structure in the form of oxygen vacancies. HRTEM reveals the existence of hexagonal layered MoS_2_ formation on the spherical shaped TiO_2_ nanoparticles at the interface. X-ray photoelectron spectroscopy recommends the chemical interactions between MoS_2_ and TiO_2,_ specifically, oxygen vacancies. In addition, the electrochemical impedance spectroscopy studies observed that L-MT sample performed low charge transfer resistance (336.7 Ω cm^2^) that promotes the migration of electrons and interfacial charge separation. The photocatalytic performance is evaluated by quantifying the rate of Congo red dye degradation under visible light irradiation, and the decomposition efficiency was found to be 97%. The electron trapping recombination and plausible photocatalytic mechanism are also explored, and the reported work could be an excellent complement for industrial wastewater treatment.

## Introduction

Heterojunction nanostructured semiconductors have been used in different applications since its discovered. The tremendous usage in a photocatalytic activity such as removal of environmental pollutants, and water splitting hydrogen technology. The photocatalytic materials have a tendency, direct energy transfer from light interaction to highly reactive chemical species^1^. Currently, heterojunction nanostructured photocatalysts are becoming more promising than the individual components because it creates a synergic effect given rise to high photocatalytic activity^2^. The main advantage of the heterostructure process is that it prevents the faster electron–hole recombination reaction and additional time for electron /or hole to reach the surface of the photocatalyst. Therefore, the rate of redox reaction increases via electron–hole enrichment. Among myriads of semiconductors, titanium dioxide (TiO_2_) is one of the well-studied traditional semiconductor photocatalysts for the removal of organic pollutants^3^. TiO_2_ is relatively non-toxic, high photostable, strong oxidizing, abundant and low-cost source material^4^. However, the principal deficiency of its absorption edge falls in the UV region at 385 nm i.e. the bandgap energy ranges between 3 and 3.2 eV^5^. The wavelength equal or less than these critical value scan retains only 3–5% of the solar spectrum. Consequently, the need for improvement in heterostructures or hybrid catalysts comes into the picture^6–10^.

TiO_2_ hybrid catalysts in different combinations are flourishing wherein MoS_2_ has drawn wide attention because of its layered structure similar to that of graphene^11,12^. 2D layered MoS_2_ comprises of the arrangement of layers stacked together with weak Van der Waals forces, in which Mo atom in the middle is strongly bonded to S atoms present above and below^13,14^. It is interesting to note that the 2D layered crystal structure offers a convenient pathway of electron transfer associated with many active sites for efficient sunlight absorption. Furthermore, MoS_2_ has astonishing advantages, including high hardness, vigorous oxidizing activity, high stability and low cost with nontoxicity^15–17^. There are captivating reported literature that focuses on synthesizing heterostructured nanocomposites such as MoS_2_@RGO, MoS_2_@TiO_2_, MoS_2_@CuO, MoS_2_/CuS, MoS_2_/MoO, MoS_2_/AgVO_3,_ TiO_2_/CdS, MoS_2_/CdS, WS_2_/TiO_2_^18–26^. In particular, MoS_2_@TiO_2_ heterostructure is an efficient photocatalyst under visible light irradiation as evident from their separation of charge carriers and active surface sites. The proposed nanocomposite has potential application for functional surfaces especially in anti-reflective surface coatings.

Li et al^27^. demonstrated that MoS_2_@TiO_2_@poly methyl methacrylate (PMMA) shows better photocatalytic performance than that of TiO_2_@PMMA and MoS_2_@PMMA. The selective deposition of (101) plane MoS_2_ nanosheet facets on (001)-TiO_2_ nanosheets enhanced the photocatalytic H_2_ production^28^. MoS_2_ nanosheets coated TiO_2_ nanorods showed two times higher photocatalytic effect than Pt@TiO_2_^29^. Another work on MoS_2_@TiO_2_ developed from protonic titanate nanosheets as precursor benefits high electrocatalytic hydrogen evolution, i.e., 26 times greater than pristine MoS_2_ based on the cathodic current density^30^. Moreover, the MoS_2_@TiO_2_ heterostructure prevents the self-aggregation of MoS_2_ that offers lattice mismatches. Pu et al^31^. developed an efficient method to remove ammonia for environmental control in agronomic livestock using MoS_2_@TiO_2_ carbon nanobelts (CNBs) demonstrating excellent photocatalytic activity in the degradation of ammonia gas with almost 91% efficiency and stability for more than a minute. Photodegradation of methylene blue showed that the MoS_2_@TiO_2_ heterojunction has a better catalytic effect up to 90% within 100 min as influenced by different nano-morphological shape impact^32^.

Industrial organic pollutants like dyes and pesticides are major crises to the environment, which are offensive discharge from industries into water resources. A huge amount of toxic organic pollutants are primarily released from textile, pharmaceutical, food, leather and cosmetic industries^33^. The highly poisonous and the non-biodegradable dyes predominantly with an azo group of dyes (–N = N–)^34^ are most commonly used in wool and paper industries as coloring agent^35^. These dyes are highly risk to health; it cause seye irritation, kidney, bladder, liver cancer, carcinogenic and genotoxic to human. Hence, tremendous care has to be taken into account for the effective handling of toxic pollutants to save the micro-organism and aquatic ecosystem^36^. We have a great responsibility to address this major challenging issue as there is much concern for researchers to find an appropriate treatment for the decomposition of Congo red dye and other dyes by an effective catalyst. The main interest is to dispose or destroy hazardous chemicals with the development of an efficient catalyst for the exclusion of toxic effluent from the environment which forms the major scope of the research established in our present work.

Herein, we are providing hexagonal 2D-layered MoS_2_ decorated on spherical shaped TiO_2_ nanoparticles for photocatalytic applications. The effects of different capping ligand binders with TiO_2_@MoS_2_ heterostructure were investigated. The photocatalytic activity of synthesized materials quantifying the rate of Congo red dye degradation in aqueous solution under visible light irradiation. The surface dependent interfacial electronic structure implies the different charge transfer behaviour between MoS_2_ and TiO_2_ heterostructures. The electron trapping recombination and a plausible photocatalytic mechanism is also discussed.

## Results and discussion

### XRD analysis

**Figure 1:**
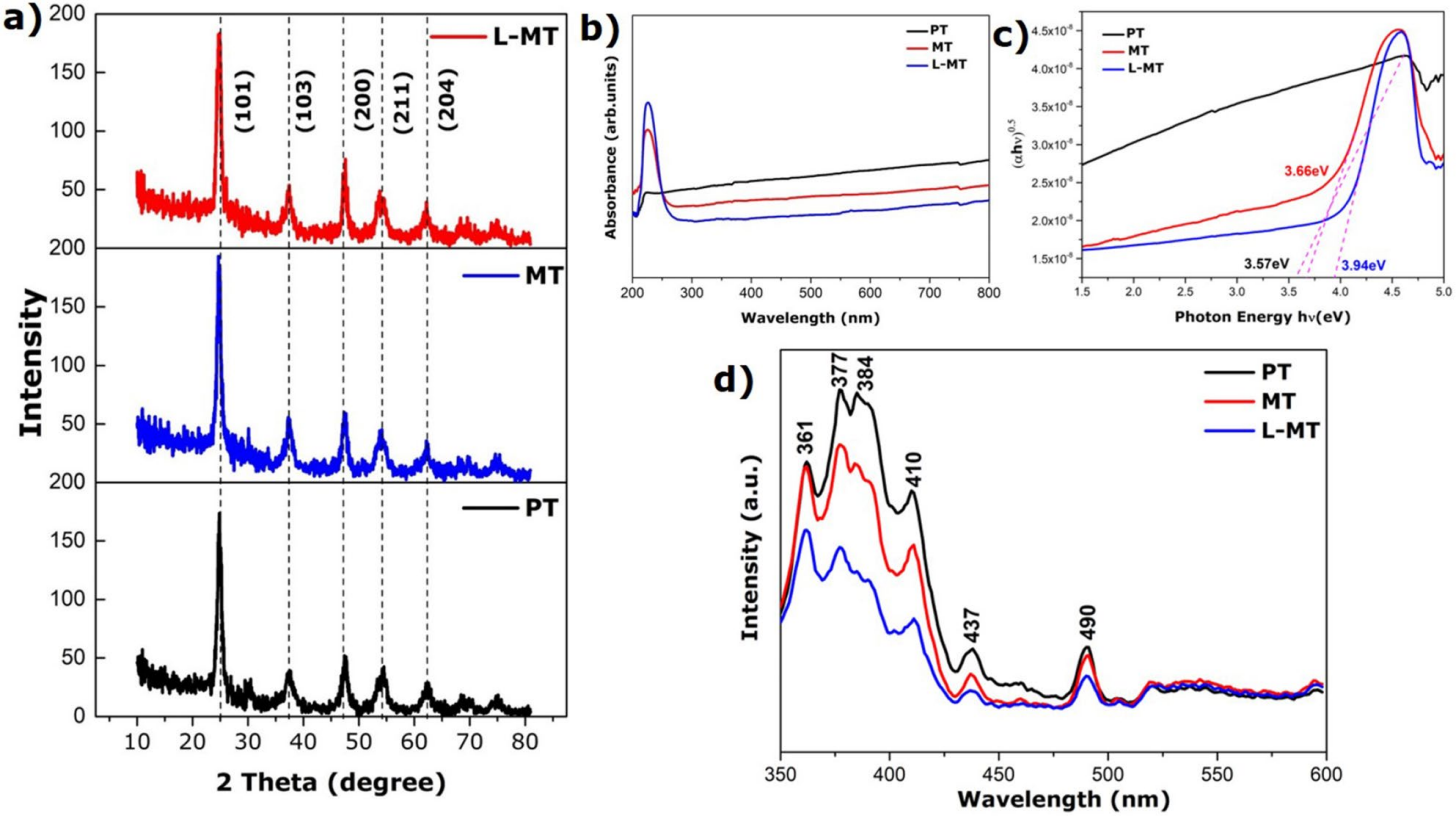
XRD patterns (**a**). UV–Vis Spectrum (**b**). Tauc plot (**c**). Photoluminescence spectrum of as-prepared PT, MT and L-MT samples (**d**).

The structural and physical analysis of the samples were studied by X-ray diffraction (XRD) using Bruker advance diffractometer with a scanning rate of 5° per min with Cuk_α_ radiation source (λ = 1.54060 Å) operating at 40 kV. Figure 1a shows the XRD pattern of prepared CTAB capped TiO_2_ within the 2θ range between 10 and 80°. The diffraction peaks located at 25.28°, 38.57°, 48.05°, 55.06° and 62.68° corresponds to the planes (101), (103), (200), (105), (211) and (204), respectively which follows the standard JCPDS pattern (21–1272). The XRD patterns of MoS_2_@TiO_2_ and L-Cysteine capped MoS_2_@TiO_2_ heterostructure has indexed as a tetragonal lattice and body centered phase of CTAB capped TiO_2_ with lattice constants as a = b = 3.785 nm, c = 9.513 nm, lattice angles as α = β = γ = 90° and space group as I4_1_**/**amd. The presence of diffraction of anatase TiO_2_ suggests that 2D-MoS_2_ nanosheets loading does not change the crystal phase of TiO_2_. On the other hand, no apparent peaks for MoS_2_ could be detected, due to its relatively lower amount along with its high dispersity and week intensity that is in well agreed with previous reports^37,38^. Further, no other impurity peaks are observed in the XRD patterns evidencing the single-phase formation of the samples. The main diffraction peak of TiO_2_ at 25.32 and 37.84° are ascribed to (101) and (103) planes, respectively^39,40^^.^ The neglectable influence of thermal reduction parades on the crystal phase and crystallinity of TiO_2_. The crystallite size was calculated using Debye Scherrer equation D = kλ/βcosθ. The crystalline size of CTAB capped TiO_2_(PT), MoS_2_@TiO_2_(MT) and L-Cysteine capped MoS_2_@TiO_2_(L-MT) are 12.6, 11.7 and 10.2 nm respectively. Interestingly, the grain surface relaxation contributes to the line broadening resulting in the reduction of the measured value of dislocation density^38^.

### Optical studies

UV–Visible spectrum of the prepared PT, MT and L-MT in the range of 200 and 800 nm is shown in Fig. 1b.The intense absorption peak is found between 200 and 275 nm in the ultraviolet region. It is evident from this spectrum that the prepared CTAB capped TiO_2_, MoS_2_@TiO_2,_ and L-cysteine capped MoS_2_@TiO_2_ nanoparticles are found to have higher absorbance in UV-region. The absorption edges of PT, MT and L-MT are estimated, and their corresponding energy band gaps are 3.57, 3.66 and 3.94 eV, respectively. The energy bandgap of the material is related to its absorption coefficient, and energy of the photon, as explained by the Tauc’s relation is shown in Fig. 1c. The increasing absorption/bandgap values show the increment of MoS_2_ on TiO_2_ surface ^9,41^. The L-MT heterostructure could be well controlled by the existence of oxygen vacancies that were introduced during the synthesis. This was established by the extent of the absorption of light from UV to visible region^42^.

The changes in the optical properties of L-MT heterostructure can be controlled by defects such as oxygen vacancies is furtherly investigated through photoluminescence spectra^43^. The spectra of the prepared PT, MT and L-MT^44^ with an excitation wavelength of 320 nm in the range between 350 and 600 nm are given in Fig. 1d. The peaks observed in the evident region are connected to the subsistence of oxygen defects in the MT, L-MT. The peaks presented at 361, 377, 410, 437, and 490 nm corresponds to the ultra-violet, violet and blue region, respectively^45,46^. The high photoluminescence intensity for PT, MT sample explored the hasty electron–hole pair recombination. Whereas, the intensity of L-MT emission peaks are found to be tuned down, due to the efficient photo-carrier separation at heterojunction interfaces^47^. Additionally, the drop in photoluminescence intensity occurs due to chemisorption of oxygen molecules leading to an increase in conductivity and it helps to avoid the recombination process^48^. The luminescence is related to the recombination of electrons in single occupied oxygen vacancies with photoexcited holes in the valance band. Photoluminescence spectra of anatase phase titania shows that the peaks present at above 400 nm is ascribed to defects in the crystalline structure such as oxygen vacancies, which also reported by He et al., Fang et al. and B. Choudhury et al^49,50^. These defects accept electrons in the photoinduced reaction with a reduction in the recombination rate of the exciton. The blue emissions peak is observed at 490 nm, and it might indicate a profound level of visible emission to localize levels in the bandgap power^42^. The sample MT and L-MT show lower intensity because of defects. These defects may leads to rarer electron–hole pair recombination possibility^51–55^. The lower intensity indicates the more efficient separation of photoinduced electrons (e−) holes (h+), thereby expecting higher photocatalytic activity^56–59^. These results demonstrate that the developed MoS_2_ hexagonal sheets have efficient light-harvesting in the visible region^60^.

### X-ray photoelectron spectroscopy (XPS) analysis

**Figure 2:**
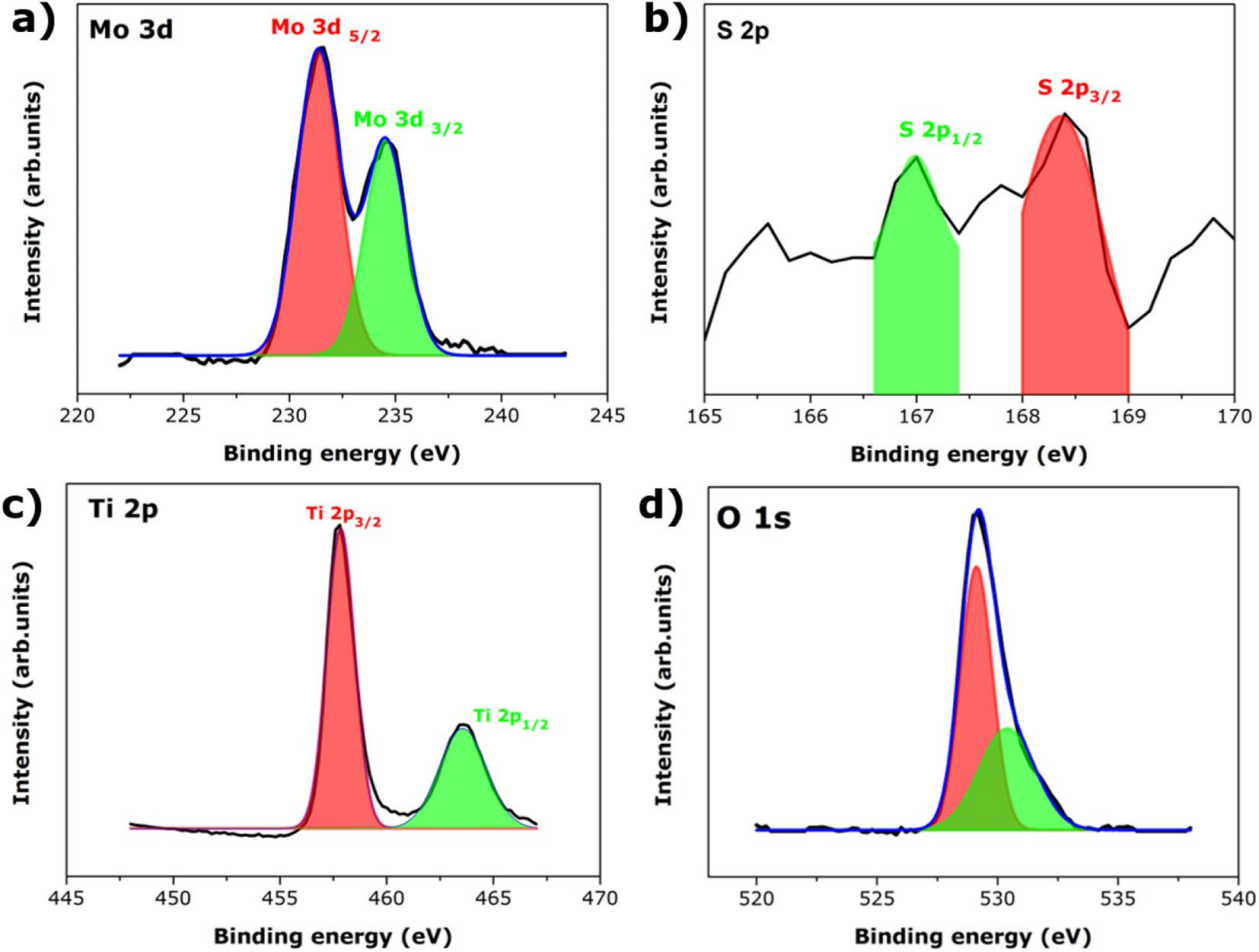
XPS spectrum of L-cysteine capped MoS_2_@TiO_2_ nanostructures (L-MT).

X-ray photoelectron spectroscopy shows the surface composed elements in L-cysteine capped MoS_2_@TiO_2_ (L-MT) nanoparticles. The characteristic peaks clearly evidence the presence of Mo, S, Ti and O elements. The high resolution XPS spectra of Mo 3d, S 2p and Ti 3p, O 1s binding energy confirms the formation of MoS_2_@TiO_2_ heterostructure. The deconvolution peaks of Mo provide information about the Mo^4+^ oxidation state and the corresponding peaks presented in ~ 231.4 eV and ~ 234.53 eV respective to Mo 3d_5/2_ and Mo 3d_3/2_ are shown in Fig. 2a with the slightly shifted peaks resulting from the composition of TiO_2_. In general, the standard energy separation difference between Mo 3d_5/2_ and Mo 3d_3/2_ is about 3.1 eV reported in literature^41^ are in well agreed with the energy difference of 3.13 eV demonstrated by our samples. The slight shift difference can be accounted to the strong interaction between MoS_2_ on TiO_2_ surface. Additionally, Fig. 2b shows two peaks at ~ 167.0 eV and ~ 168.5 eV confirms the presence of sulphur that could be correlated to S2 p_1/2_ and S2 p_3/2_ confirming the formation of MoS_2_. The energy separation between 2p_1/2_ and S 2p_3/2_ is 1.1 eV and agrees well with the reported values^41^. TiO_2_ peaks are also observed at around ∼457.7 eV (Ti 2p_3/2_), ∼464.0 eV (Ti 2p_1/2_) as seen in Fig. 2c and O peaks are seen in Fig. 2d. The increasing energy state from ~ 529.4 to ~ 530.53 eV correlates to O1s peaks. XPS measurement inveterate the presence of TiO_2_ the as-prepared heterostructure. All the samples showed significant peaks shift because of the strong interaction between Mo and Ti.

### Morphology (HR-TEM) and EDAX spectrum image analysis

**Figure 3:**
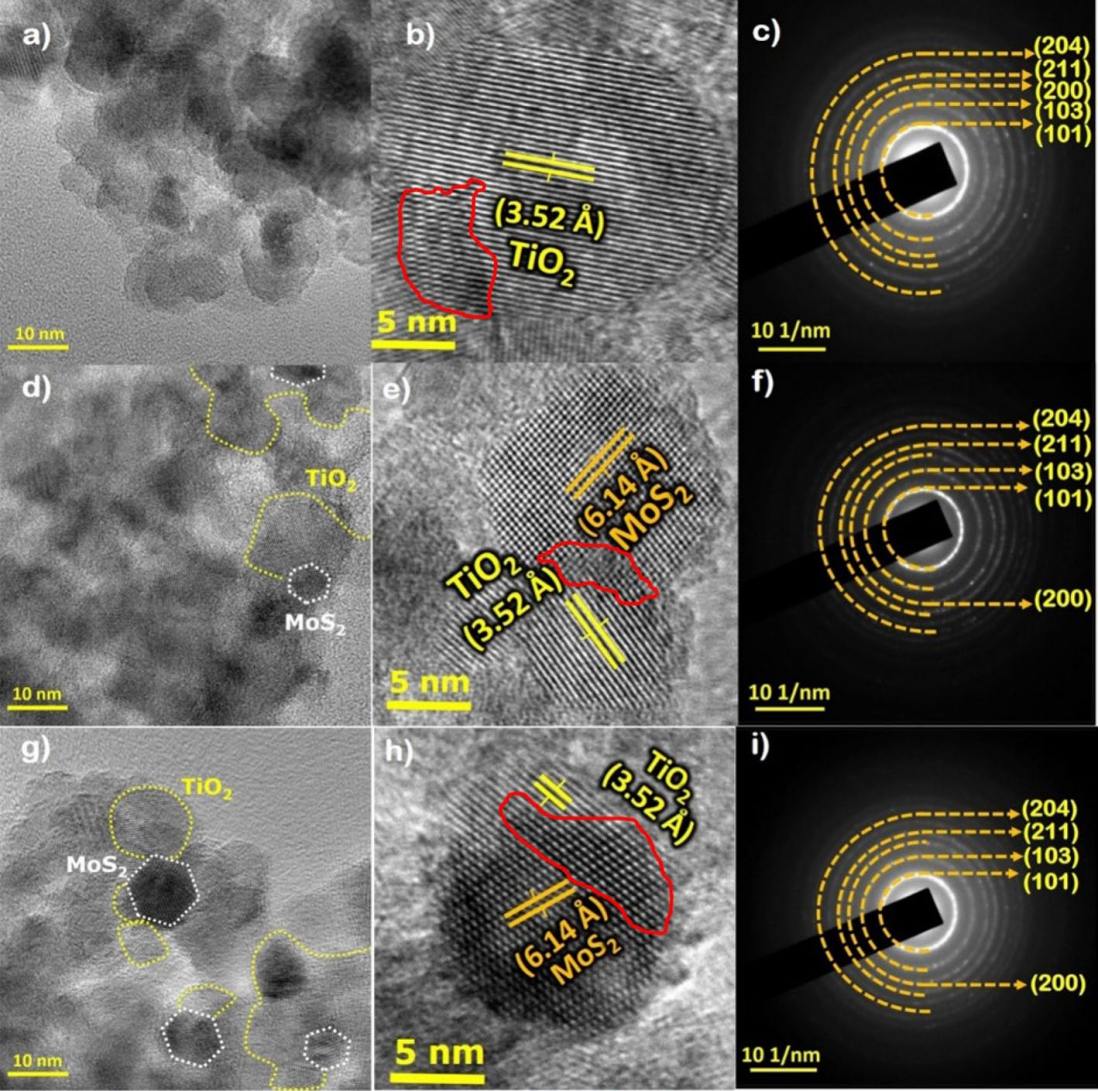
TEM images, HRTEM and SAED pattern of PT (**a**–**c**). MT (**d**–**f**). L-MT samples (**g**–**i**).

The morphology of the as-prepared PT, MT and L-MT heterostructures are examined through JEOL 2100 instrument with an operation voltage of 25KeV. All three images depicted in Fig. 3a–i displayed very interesting morphology with clear edge site overlaps between TiO_2_ and MoS_2_. Herein, we have found that the hexagonal layered MoS_2_ are attached to the spherical-shaped TiO_2_ nanostructure. The specific HRTEM images of (Fig. 3b,e,h) confirm the obvious observation of layers on spherical or vice versa. It confirms the decoration of the surface in TiO_2_ nanoparticles with thin MoS_2_ nanosheets. This identifies the elemental composition and EDAX spectrum of Mo, S, Ti, O distribution (see Supplementary Fig. S1 online).

**Figure 4:**
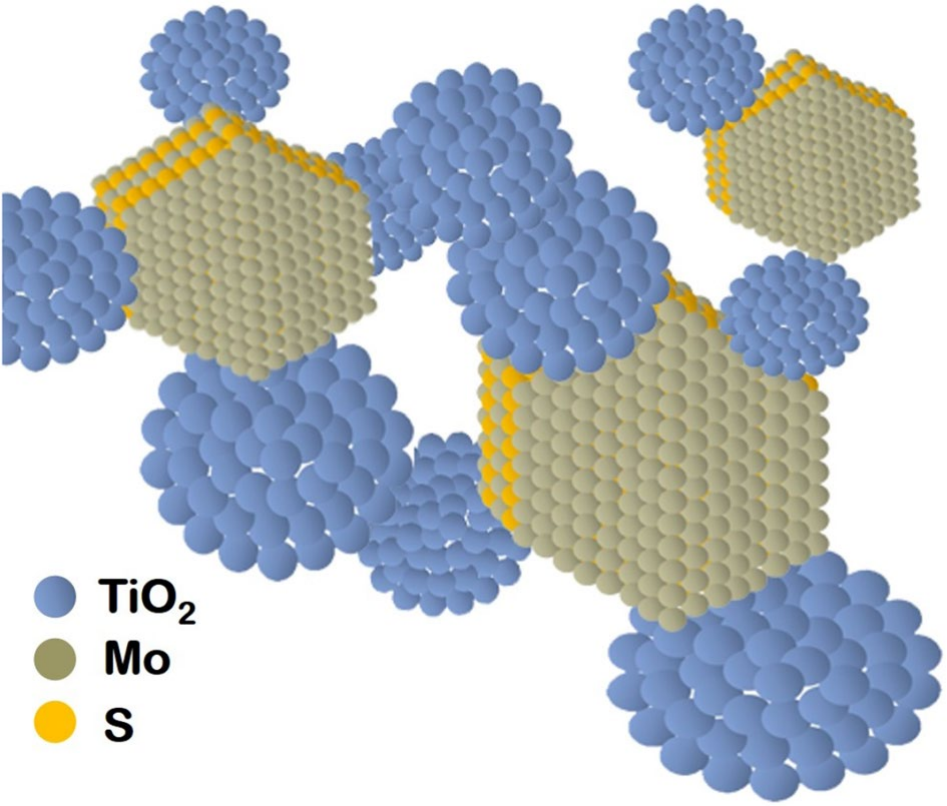
Schematic representation of hexagonal layered MoS_2_ attached on spherical shaped TiO_2_.

Figure 4, represents the hexagonal layered MoS_2_ decorated on spherical shaped TiO_2_ nanoparticles. It is interesting to emphasize that most of the nanosheets and nanoparticles are overlapped towards the edge site. The HRTEM images of the heterostructure display two kind of lattice fringes as shown in the heterostructure. The attachment between MoS_2_ and TiO_2_ nanoparticles well aggregate the interparticle adhesive nature^61^ with few coarsening as seen from the observed irregular profile that might be attributed to the thermal flux effect of heterostructures synthesis. The selected area electron diffraction (SAED) pattern suggests the existence of numerous ring patterns to explore the crystalline nature of the synthesized MoS_2_ nanosheets on the TiO_2_ nanostructure in detail.

HRTEM observation revealed a greater number of MoS_2_ nanosheets grown on the surface of TiO_2_ nanoparticles together with the minimal observation of an elevated aggregation level of the heterostructure. The HRTEM images presented in Fig. 3d of MT disclose two kinds of lattice fringes confirming the presence of 5–6 individual layers. Furthermore, HRTEM images are given in Fig. 3e,h shows a discontinuous area at the interface between MoS_2_@TiO_2_ which clearly indicates the presence of oxygen defects in the structure. The large separation seen between TiO_2_ and MoS_2_ nanosheets also poses defects resulting from their lattice mismatch. The crystal interface between MoS_2_@TiO_2_ shows a distorted atomic pattern and significant lattice distortions, which consequences in a change of periodicity and the formation of structural defects. This interface improves the photoinduced charge carrier transfer and significantly increases the number of active catalytic sites^62–65^. Densely packed disordered areas have been observed around the interface because of lattice stress and robust interfaces between MoS_2_ and TiO_2_.

This is consistent with photoluminescence spectra arguments as stated above. The nanosheet grown on the surface of TiO_2_ with lattice spacing of 6.14 Å correlates to the (001) plane of MoS_2_. The set of major fringes spacing measured as 3.52 Å could be related to the (101) lattice spacing of anatase TiO_2._ The lattice fringes are well matching with previous research work^61^.

Similarly, XPS spectrum (Fig. 2) displays the shifting of Ti_2P_ peaks to the lower binding energies after the deposition of TiO_2_ onto MoS_2_ nanosheets. The above-mentioned result evidence that titanium (Ti) atoms accept electrons from MoS_2_, resulting in increased Ti^3+^ in the heterostructures. The coupling of MoS_2_ with (101) faced TiO_2_ results in a totally different change of spectra. The shift of Ti2p peaks to higher binding energies indicates the functioning of Ti atom as an electron donor. The surface dependent interfacial electronic structure implies the exchange charge transfer behaviour between MoS_2_ and TiO_2_ heterostructures^37^.

Moreover, the charge carriers in semiconductor photocatalyst strongly depends on the exposed facets and structural defects. Consider the example of (001) and (101) facets/phase of TiO_2_, two most common facets of anatase phase, usually exhibit different adsorption characteristics and redox abilities during the photocatalytic reaction^37,66,67^.

In the present work, its directly observed that the HRTEM images of 3b,e,h have similar characteristic facets of (001) and (101). It indicates the exposure of (101) facets as more favourable for the formation of surface oxygen vacancies. The marked red coloured circles of HRTEM images represent the actual defects sites. This defect modulation is an effective strategy that helps to improve photocatalytic activity of (101) faced TiO_2_ molecules. In order to study the synergetic effect between oxygen defects and MoS_2_, the charge carrier’s behaviour was studied by PL measurements. Obviously, the deposition of MoS_2_ and oxygen vacancy formation results in the PL emission of (001) and (101) faced TiO_2_^37,68,69^. Eventually, the mid gap between MoS_2_ and TiO_2_ oxygen defects states can act as an electron mediator to facilitate this charge transfer^37^.

PL peaks at 361, 377, 410, 437, and 490 nm correspond to oxygen defects. In general, the PL spectra of anatase phase TiO_2_ nanomaterials are attributed to three kinds of physical origins: (1) self-trapped excitons^70,71^ (2)oxygen vacancies (OVs) ^71,72^ (3) surface states^73^. In the TiO_2_, Ti^4+^ ions adjacent to oxygen vacancies^74,75^. Few reports also documented PL properties of anatase phase single crystals evidencing self-trapped excitons localized on TiO_6_ octahedra^3,71^.

The peak position of 410 nm established in the current work corroborates well with the emission band at ~ 412 nm referred in the previous literature^3^. Hence, it is clearly evident that 412 nm band was assigned to self-trapped excitons localized on TiO_6_ octahedra^3^. The PL bands at the long wavelength side of anatase TiO_2_ nanoparticles have been attributed to the oxygen vacancies(OVs)^76^. The OVs sites were occupied by O_2_^-^ ions in the -Ti–O network and are ascribed to *F*^+^ centres^76^. Furthermore, the emission peak centred at 437 nm is also very close to 433 nm as given in the reported literature which could be assigned to self-trapped exciton^52^.

In a similar manner, the peaks position at 490 nm is also much related to the reported value of 492 nm^52^. This peak position occurs charge transfer transition from Ti^3+^ to TiO_6_^2-^ complex and are well associated with oxygen defects^77,78^. The efficiency of the PL emission is accounted to both radiative and non-radiative recombination process.

### Visible light induced degradation of Congo red using L-MT heterostructure

**Figure 5:**
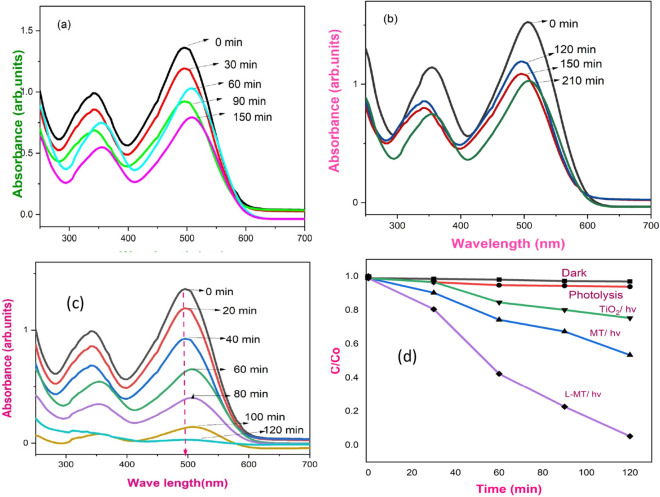
UV–Visible absorption spectra of CR dye with PT (**a**). MT (**b**). L-MT (**c**) (Reaction conditions: dye concentration 15 mg/L, pH3/60 mg cat); C/Co vs irradiation time (**d**).

The degradation of highly carcinogenic effluents such as Congo red dye was explored using the prepared PT, MT and L-MT as given in Fig. 5a–c. These azodic groups of dyes are highly carcinogenic and genotoxic to human health causing various diseases. Hence, the photocatalytic decomposition of Congo red was examined with the irradiation of visible light (λ = 400 nm) via L-MT heterostructure^31,33^, which shows the higher degradation nature among all the three samples. The well-arranged crystalline structure with more active surface area and smaller size effect of the hexagonal layered MoS_2_ attached on the spherical shaped TiO_2_ nanoparticles in L-MT samples leads to excellent decomposition nature than that of the other two samples. The HRTEM images (Fig. 3b,e,h) corroborate the layers on spherical or vice versa. Eventually, the surface of TiO_2_ nanoparticles was decorated with thin MoS_2_ nanosheets is a major source of more reactive sites. The enduring destruction and increased removal capacities of harmful chemicals using L-MT were found to be more due to its shapes, porous and crystalline nature.

**Figure 6:**
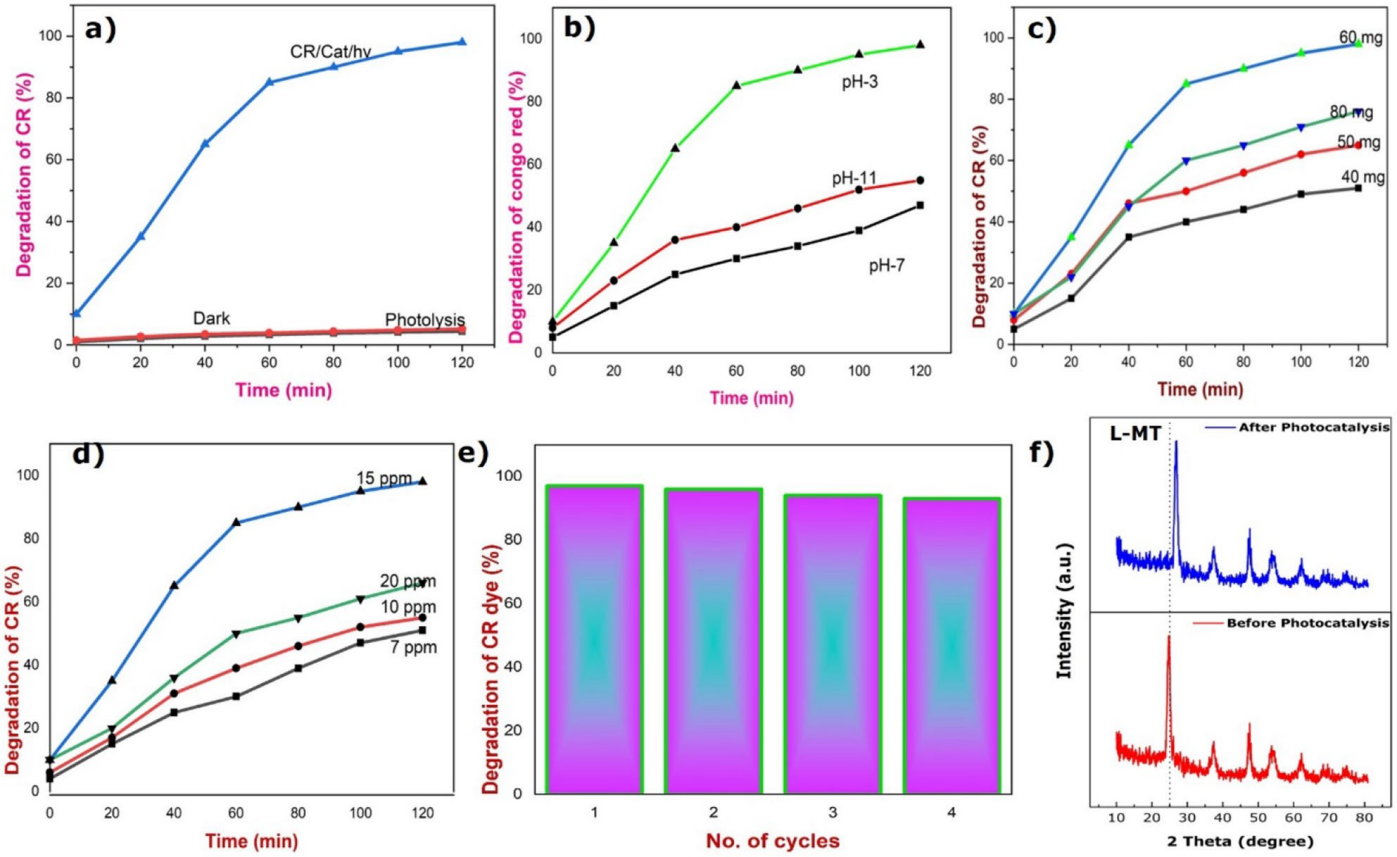
Degradation of CR dye (%) versus irradiation time. (Reaction conditions: dye concentration 15 mg/L, pH 3/60 mg L-MT catalyst) (**a**). Degradation of CR dye with the pH (Reaction conditions: dye concentration 15 mg/L, 60 mg cat) (**b**). Degradation of CR dye by tuning catalyst concentration (Reaction conditions: dye concentration 15 mg/L, pH3) (**c**). Degradation with different strength of CR dye (Reaction conditions: (pH 3/60 mg cat) (**d**). Reusability of L-MT hetero-junction photocatalyst (Reaction conditions: dye concentration 15 mg/L, pH 3/ 60 mg cat) (**e**). XRD analysis of photocatalyst before and after photocatalytic reaction (**f**).

The absorbance spectra of CR dye (15 ppm) during the decomposition process with 60 mg of catalyst on the illumination of visible light at pH-3 is given in Fig. 5c. The absorption spectra show the two peaks at 350 nm and 495 nm corresponding to the aromatic ring and π–π* transitions emerging from the azodic group. The decomposition of Congo red dye was proved by the reduction in peaks intensity and color change from red to a colorless solution with increasing the time interval under visible light irradiation^79,80^. Similarly, the above-mentioned experiment was carried out in the absence of the photocatalyst and in the dark condition as shown in Fig. 5d. In addition, the same experiment is also carried out in neutral and basic medium by the addition of HCl/NaOH which differ in the time of decomposition. The plot between C/Co and time in a minute is portrayed in Fig. 5d. Furthermore, the decomposition efficiency of Congo red dye was shown in Fig. 6a and found to be 97% in the acidic medium with 120 min of irradiation of light source, whereas the degradation was taking a longer time duration in the basic medium^81–84^. The complete mineralization of the CR was attained within 120 min in acidic conditions. To conclude, the above results of the synthesized photocatalyst holds extensive photocatalytic activity not only for Congo red and have great potentiality for decomposing other colored dyes too^85–91^.

### Effect of pH on the photocatalytic activity of L-MT

The degradation efficiency of L-MT heterostructure on photodecomposition of CR is detailed for a range of pH from 3 to 9 to examine the effect of pH. Figure 6b is evidencing that the pH drastically pretentious the rate of degradation of CR. The rate of reaction decreased from 97 to 46% with the change in pH value from 3 to 11 upon illumination of visible light with a time interval of 120 min. The highest rate of reaction is obtained for the degradation of CR nearly 97% at pH3. The result of the degradation process of CR indicates the surface charge of catalyst as determined by the activity of ions (e.g. H^+^) in different medium. The catalyst surface becomes either positive or negative charged with the function of pH. The synthesized L-MT heterostructure could follow the process of association or dissociation. Subsequently, it affects the degradation processes as well as the generation of exciton and migration of the photoinduced electron–hole pairs at the surface of the catalyst. The decomposition efficiency is nearly 97% in the neutral and basic conditions after 5 h and 8 h, respectively^81^.

In acidic medium, the reaction mixture consists of more protons than hydroxide groups; implies that the positive charge is increased on the surface of L-MT heterostructure which in turn results in an attraction of the anionic dye with the positive surface of the catalyst. As a result, complete decomposition occurs with a minimum duration of nearly 120 min. When the pH is above 7, the decomposition desires longer time for the degradation of CR. The rate of decomposition of anionic CR dye is greater in acidic medium as compared with basic and neutral medium. Consequently, the adsorption and decomposition of CR dye on the catalyst on illumination of visible light becomes higher in acidic medium. Thus, the decomposition process revealed that the catalyst has a great ability to be used as an efficient catalyst for the various industrial effluents. The electrostatic interaction occurs between the negatively charged dye and the positively charged surface of the catalyst in the acidic environment. In the case of basic condition, the heterogeneous catalyst carries a negative charge on the surface with the intention of repulsions by the anionic dye solution, thereby decreasing the decomposition of dye. Furthermore, the decomposition is possible in different pH due to hydrogen bonding, hydrophobic–hydrophobic interactions and Van der Walls forces etc.^81^, The mechanism of degradation in acidic and basic medium can be well-understood from the following equation:

In acidic medium,

$${\text{MoS}}_{2} - {\text{TiO}}_{2} + {\text{H}}^{ + } + {\text{CR}}^{ - } \to {\text{MoS}}_{2} - {\text{TiO}}_{2} + {\text{H}}^{ + } + {\text{CR}}^{ - } \left( {{\text{electronic}}\;{\text{attraction}}} \right)$$

In basic medium,

$${\text{MoS}}_{2} - {\text{TiO}}_{2} + {\text{OH}}^{ - } + {\text{CR}}^{ - } \to {\text{MoS}}_{2} - {\text{TiO}}_{2} + {\text{OH}}^{ - \cdot } \ldots {\text{CR}}^{ - } \left( {{\text{electronic}}\;{\text{repulsion}}} \right)$$

### Effect of catalytic dose in the photo-decomposition

To evaluate the effect of the amount of catalyst in the decomposition of CR, the experiment was conceded with the various amount of catalyst from 40 to 80 mg. The intensity of visible light source, the concentration of CR dye solution (15 ppm) and pH of the solution remains constant in the degradation process. The catalytic dose is increased from 40 to 60 mg in dye solution with increasing decomposition rate of the reaction as evidenced from Fig. 6c However, the degradation is reduced by raising the concentration of catalyst above 60 mg due to the scattering of light and poor penetration of light in the reaction mixture. When the catalytic amount increased gradually in the reaction mixture, the energetic molecules of the catalyst reduced owing to the aggregation of catalyst that further leads to turbidity causing subsequent minimization of the dispersion of light in the reaction medium. As a result, the complete catalytic decomposition of CR dye can be achieved with the use of 60 mg as a suitable catalytic dose.

### Effect of initial dye concentration

The strength of the industrial effluent like CR dye plays a vital role in the photodecomposition process. Hence, the effect of initial strength of dye solution in the reaction process was examined with different strength of CR dye solution (7, 10, 15, 20 mg/L) as presented in Fig. 6d. During this decomposition process, the dose of catalyst and pH of the reaction mixture remains constant throughout the overall process. The strength of the dye solution is found to increase from 7 to 15 mg/L with an increase in the efficiency of decomposition of CR dye. However, the rate of decomposition of CR dye is observed to decrease corresponding to the strength of dye solution greater than 15 mg/L, which confirms the fact that the removal of CR dye depends on the initial strength. Further, with the increase in the concentration of CR dye, the degradation underway to decrease^82^.

### Reusability of L-MT heterostructure photocatalyst

In order to identify the reusability nature of the used catalyst after the photo degradation reaction, the used catalyst was removed from the reaction mixture, washed with distilled water and dried in the oven at 120 °C to further estimate its reusability. The reusability and consistent nature of the used L-MT catalyst after the photodegradation reaction was tested with four cycles by removing the catalyst from the reaction mixture. At the end of the last cycle, the decomposition efficiency was found to be 95% under illumination of visible light as seen in Fig. 6e Furthermore, Fig. 6f proves that the occurrence of typical peaks in the XRD patterns for the L-MT sample after fourth cycle. In addition, the good quantity of used catalyst was separated from the reaction mixture at the end of fourth cycle. The physical property of the fresh and used photocatalyst remains almost the same, which is also evidence for the decomposition of CR and the absence of adsorption of dye on the photocatalyst. The well-established stability of the catalyst increases its practical usage as a catalyst in photo decomposition of CR^49^. Thus, the as-synthesized L-MT sample was stable and reusable. Consequently, the results corroborate the stability of the catalyst and its reusability for long duration.

### Electrochemical impedance spectroscopy studies

**Figure 7:**
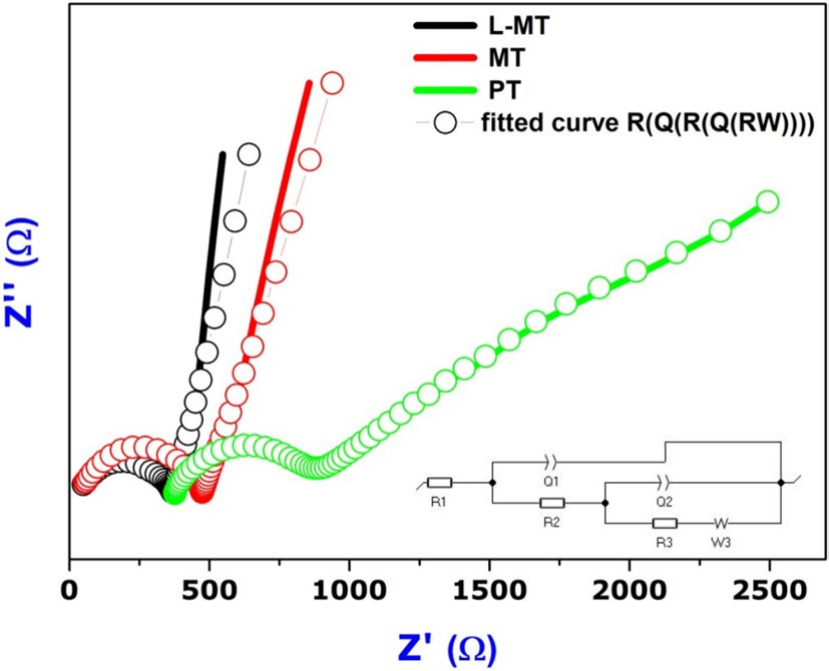
Electrochemical impedance spectroscopy of PT, MT and L-MT.

The working electrodes prepared by the as-synthesized samples (PT, MT and L-MT) consists of 95 mg of sample along with 5 mg of polyvinylpyrrolidone (PVDF) ground in 1 ml of N-methy l-2-pyrrolidone (NMP) and the resulting slurry was later coated on an FTO plate and dried for 12 h at 80 °C. Nyquist plot shown in Fig. 7 of the as-synthesized samples PT, MT and L-MT explains the charge-transfer between the working electrode and the electrolyte solution. The electron transfer resistance controls the charge kinetics at the electrode interface and is totally dependent on the diameter of Nyquist circles.

A tiny diameter concludes the exaggerated separation and transfer of carriers^39^. Considering the results of PT, Nyquist semicircle starts from 378 Ω and the diameter is much larger than MoS_2_ doped TiO_2_ (MT, L-MT). On the other hand, Nyquist semicircles of MoS_2_ doped TiO_2_ shifted nearly to 0. The diameter of the semicircle of L-MT is comparatively smaller than that of the other two samples thereby evidencing the increase in the separation of photogenerated electrons and holes^10^. The synthesized heterostructure nanosurface (L-MT) improved the separation efficiency of the photoexcited charge carriers. The electron transfer occurred from the conduction band of light-activated TiO_2_ to the conduction band of MoS_2_; conversely, hole transfer could take place from the valence band of MoS_2_ to the valence band of TiO_2_^83^.

The summary of EIS studies seen in Table. ST1 (supplementary table) shows the resistance and capacitance values of equivalent circuit for PT, MT, and L-MT. Charge transfer resistance (R_CT_) values are 488.2, 426.8 and 336.7 Ω cm^2^ corresponding to PT, MT, and L-MT, respectively. Interestingly, this shows that L-MT has very low charge transfer resistance as it promotes migration of electrons and interfacial charge separation together with efficient reduction of exciton quenching and energy dissipation. As a result of this process, the as-synthesized heterostructure nanosurface (L-MT) provides good degradation efficiency against Congo red dye^59^.

### Photodegradation of CR mechanism

**Figure 8:**
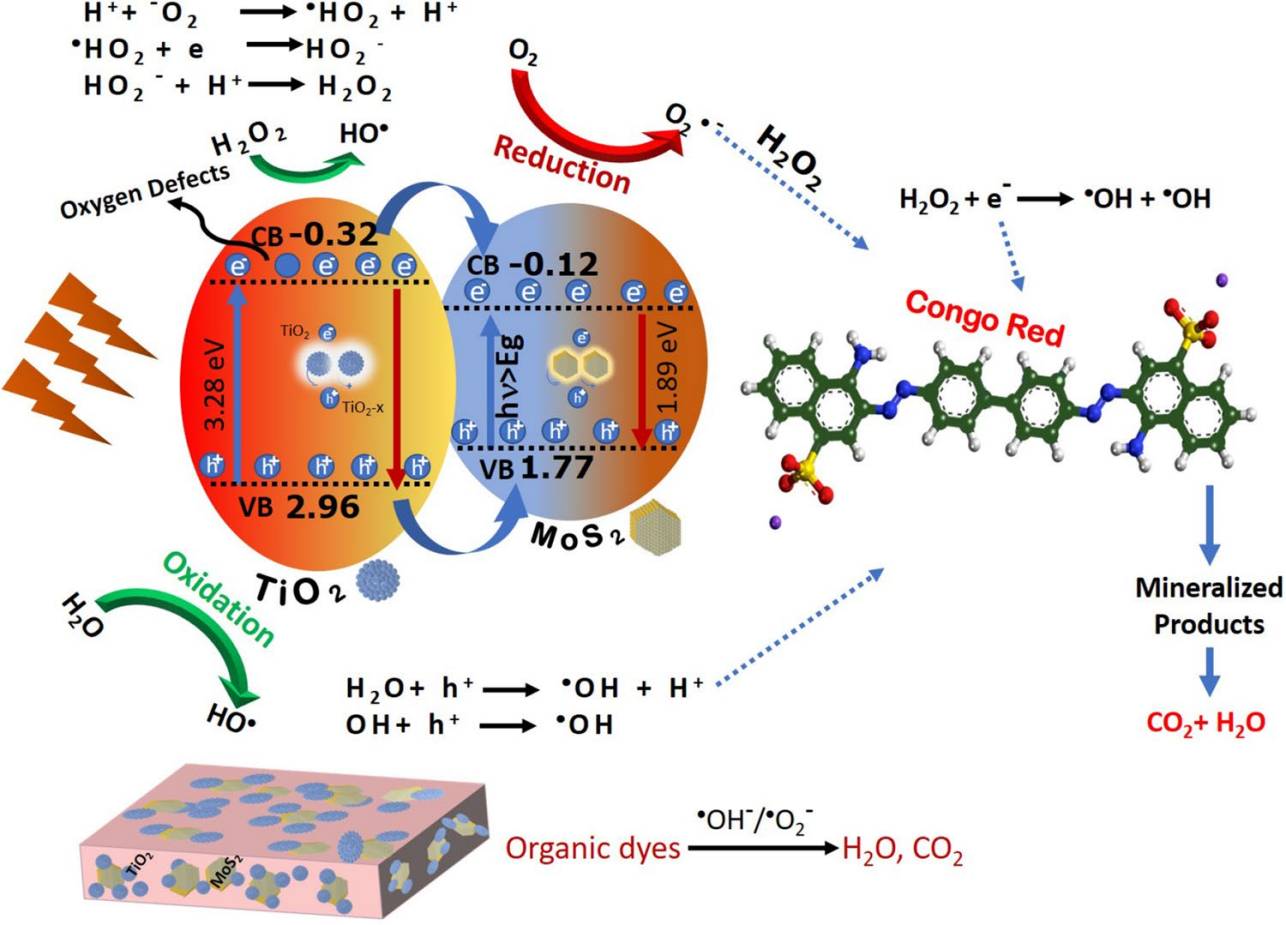
Possible schematic mechanism of photocatalytic electron trapping process in 2D-layered MoS_2_ decorated on spherical shape TiO_2_ under visible-light irradiation.

The degradation of major industrial effluents such as CR dye was enhanced by the surplus generation of excitons on the surface of the catalyst under irradiation of visible light source. The electrons in the ground state of the surface of the L-MT catalyst promotes to excited state under visible light^9,10,50^, the possible schematic mechanism shown in Fig. 8. The charge separation occurs in the valence and conduction band of the catalyst. Hence, the excited electrons by absorbing photons occupy the conduction band (CB) by leaving the holes in the valance band (VB) of the catalytic surface. In the PL spectra of PT (pristine TiO_2_) and L-MT sample, the intensity of L-MT sample is lower than that of the PT^58,92^. These observed results confirm that the L-MT heterostructure possessing more competent charge carrier separation, which leads to suppression of the exciton recombination in hexagonal 2D-layered MoS_2_ decorated on spherical shaped TiO_2_ heterostructures^84^.

**Figure 9:**
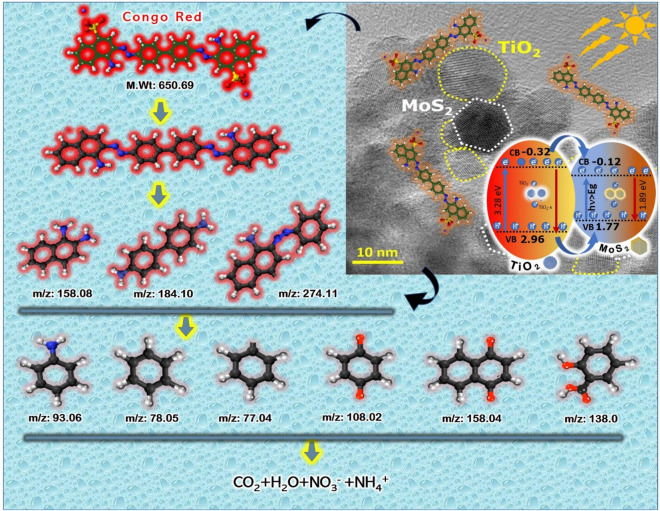
Mechanism of photocatalytic decomposition of Congo red dye in 2D-layered MoS_2_ decorated on spherical shape TiO_2_ under visible light irradiation.

In a similar manner, Congo red dye absorbs high energy photons from visible light through photosensitization progression and undergoes autooxidative revolution giving rise to circumlocutory creation of oxidizing hydroxyl (OH^·^) radicals. The electron in the highest occupied molecular orbital (HOMO) shifted to lowest unoccupied molecular orbital (LUMO) of the CR dye on visible light irradiation. The photo induced electron in the excited CR* was migrated to catalyst surface by leaving CR*+ dye to strengthen the generation of exciton. Furthermore, CR*+ reacts with active hydroxide radicals to form smaller fragments product via breaking of ring structure as shown in Fig. 9. The mechanism implicated in the progression of photosensitization is given in below Eqs. (4) and (5).

X. Zhang et al. reported the delocalization of electrons with reduction in the recombination electron–hole pairs leads to higher catalytic activity due to the presence of surface defects on the nanorods^93^. The EIS spectra confirm the more efficient separation of photoinduced electron–hole pairs and rapid interfacial charge transfer for the L-cystine MoS_2_ doped TiO_2_ heterojunction surface than the other two samples. These generated excitons position in the VB and CB of L-MT catalyst plays a vital role to increase the photocatalytic efficiency of the L-MT catalyst^51^. The reduction and oxidation of congo red in aqueous reaction mixture solution were carried out through the excitons, ensuing in significant enhancement in the catalytic performance. The band gap of TiO_2_ and MoS_2_ are 3.28 eV and 1.89 eV which is well consistent with many reported work^94,95^. The VB and CB edge position is concurrence with their electronegativity^96^. The CB and VB potentials of semiconductors are calculated using the following empirical equations:

1 $$CB = \chi - E_{e } + 0.5\,E_{g}$$

2 $$VB = CB + E_{g}$$

E_e_ is the energy of free electrons versus hydrogen (4.5 eV). Finally, χ is the electronegativity of semiconductor and it was calculated by the following equation:

3 $$\chi = \left[ {\left( A \right)_{a } x\left( B \right)_{b } x\left( c \right)_{c } } \right]^{{1/\left( {a + b + c} \right)}}$$

In which a, b, and c are the number of atoms in the compounds. The generated excitons position in the VB and CB of L-MT catalyst plays a vital role to increase the photocatalytic efficiency of the L-MT catalyst^51^. These excitons assist the reduction and oxidation of Congo red in aqueous reaction mixture solution, ensuring in significant enhancement of the photocatalytic performance. In L-MT sample, VB is to be found at 2.96 eV and CB is at − 0.32 eV for titania and MoS_2_ VB (1.77 eV) / CB (− 0.12 eV) against normal hydrogen electrode(NHE)^26,38^. The aforementioned result confirms the CB edge of titania to be less negative than that of the redox potential of O_2_/ O_2_^·−^(− 0.33 V). This process slows down the electron in the conduction band reacts with oxygen molecule to form superoxide anion radicals (O_2_^·−^)^24,32^. The electron in the conduction band must be transferred to CB of MoS_2_ that has to be used by H_2_O_2_ to generate more OH^·^ radical which is involved in the decomposition of CR dye^29,97^. The hole (h^**+**^) with higher oxidation potential can contribute to the oxidation of the CR dye^27^. The VB edge of catalyst is greater than the redox potential of OH^·^/OH^−^ (1.99 V). These positive holes are required to oxidize water to form a OH^·^ radical leading to oxidation of CR dye solution into non-toxic products like H_2_O, NO_3_^−^, NH_4_^**+**^ and CO_2_ etc^31,32,36^. The resultant generated oxidizing hydroxyl (OH^·^) radicals and reducing superoxide anions facilitates the complete decomposition of organic contaminants, as reported in previous litreatures^98,99^. In the case of MoS_2_, the mechanism procedure is followed by the generation of ROS for the further degradation of CR dye. The oxygen vacancies accept electrons in the photoinduced reaction with the reduction in recombination rate of exciton as evidenced in photoluminescence spectra. The presence of oxygen vacancies in the L-MT sample plays an imperative task for the efficiency of degradation of dye. These oxygen vacancies on the surface level of the catalyst are accountable for trapping the electron from the conduction band and play down the excitonic recombination resulting in a superior photoinduced catalytic effect.

The reactive oxygen species (ROS) generated in the reaction decomposes the CR dye into smaller units. Therefore, in the L-MT heterostructure, the recombination of excitons is reduced, further generating the strong oxidative radicals for the degradation^80^. The various parameters like pH, catalytic dose and initial concentration of dye on the photocatalytic decomposition process via L-MT heterostructure reveal the adsorption capability and high destruction performance in the degradation of Congo red. When oxygen vacancies are debuted into the L-MT, the defects can act as an electron facilitator to assist the charge transfer and separation of photoinduced electron–hole pairs^37,100^. The synergetic effect between oxygen vacancies, crystal surfaces and narrow bandgap leads to significant photocatalytic activity^60,101^. The obtained results from optical and EIS analysis are in good agreement with the results of the photocatalytic efficacy. Consequently, the generation of photoinduced excitons will be influenced by the internal electric field in the heterostructures. Hence, evidently the present work revealed that this stable catalyst in future potentiality can act as an efficient photocatalyst for environmental wastewater treatment and its purification. Moreover, the present nanocomposite with subsequent functionalization has futuristic scope for antireflective coatings too. The synergetic effect of MoS_2_ nanosheets and TiO_2_ nanoparticles results in a large number of reactive sites and poor exciton recombination for adsorption followed by decomposition^102^, enhance the photocatalytic nature. Table.ST2 (Supporting information) shows the evaluation of synthesised photocatalyst with other photocatalysts that recently used for degradation of dyes. The photo-decomposition of CR dye mechanism of L-MT heterostructures has been projected as follows:

4 $${\text{Congo}}\;{\text{red}}\left( {{\text{CR}}} \right) + {\text{h}}{{\upsilon }} \to {\text{CR}}^{*}$$

5 $${\text{MoS}}_{2} + {\text{CR}}^{*} \to {\text{CR}}^{ \cdot } + {\text{MoS}}_{2} { }\left( {{\text{e}}^{{ - {\text{CB}}}} } \right)$$

6 $${\text{MoS}}_{2} + h\upsilon \to {\text{MoS}}_{2} \left( {e^{ - CB} + h^{ + VB} } \right)$$

7 $${\text{TiO}}_{2} + {\text{h}}{{\upsilon }} \to {\text{TiO}}_{2} \left( {e^{ - CB} + h^{ + VB} } \right)$$

8 $${\text{MoS}}_{2} \left( { h^{ + VB} } \right) + {\text{H}}_{2} {\text{O}} \to {\text{MoS}}_{2} +^{ \cdot } {\text{OH}} + H^{ + }$$

9 $$\left( { h^{ + VB} } \right) + OH^{ - } \to^{ \cdot } {\text{OH}}$$

10 $${\text{CR}}/{\text{CR}}^{ \cdot - } + \left( {{\text{H}}^{ + } + {}^{ \cdot }{\text{OH}} + {\text{O}}_{2}^{ \cdot - } } \right) \to {\text{Less}}\;{\text{toxic}}\;{\text{derivatives}} \to {\text{H}}_{2} {\text{O}} + {\text{CO}}_{2} + {\text{NO}}_{3}^{ - } + {\text{NH}}_{4}^{ + }$$

## Conclusion

In summary, the present study demonstrates the novel MoS_2_ nanosheets decorated on spherical shaped TiO_2_ heterojunction photocatalysts prepared through hydrothermal approach for photocatalytic degradation of Congo red dye in visible light. It is evident from HRTEM analysis that the attachment between MoS_2_ and TiO_2_ nanoparticles well aggregated the interparticle adhesive nature. The influence of capping ligand binder on TiO_2_@MoS_2_ heterostructure was investigated. Moreover, based on the EIS analyses, the diameter of a semicircle of L-MT is very smaller that indicates the increase in separation of the photogenerated electrons and holes on the surface of L-MT heterostructure. The L-MT heterostructure exhibits strong adsorption ability and high photocatalytic performance in the degradation of Congo red that obviously revealed its future potentiality as an efficient photocatalyst for environmental applications. We have clearly outlined the effects of pH, catalytic dose and initial concentration of dye on the photocatalytic degradation process. The enhancement in photocatalytic activity of the proposed heterostructured photocatalyst is ascribed to the complementing synergetic effects of MoS_2_ nanosheets on TiO_2_ nanoparticles resulting in a large number of active sites for adsorption. From our study, it is well proved that L-cysteine capped MoS_2_@TiO_2_ heterostructure have better removal of Congo red with minimum 120 min with maximum efficiency of 97%.

## Methods

All the chemicals were used as-received and without further purification. The absolute ethanol (99.99%) was obtained from Merck chemicals Ltd. Titanium isopropoxide, cetyltrimethylammonium bromide (CTAB), potassium iodide (99.9%), citric acid, thiourea, L-cysteine and ammonium heptamolybdate were purchased from Aldrich. The entire synthesis process was performed using deionized water.

### Synthesis of CTAB capped TiO_2_ nanoparticles

The preparation of CTAB capped TiO_2_ nanoparticles was performed by sol–gel method where 2.87 mL of titaniumisopropoxide was mixed in the CTAB solution. 3.64 g of CTAB was dissolved in the mixture of 25 ml of absolute ethanol and 100 ml of deionized water (1:4 volume ratio) and the solution was stirred for 1 h to form a clear solution. Subsequently, titanium isopropoxide was added drop wise in the CTAB solution with vigorous stirring for 24 h. The resulting gel was centrifuged and washed several times with ethanol.The final product was calcined at 400 °C for 3 h in static air and the collected sample was designated as pure TiO_2_ (PT).

### Synthesis of MoS_2_ doped TiO_2_ nanoparticles

The simple co-precipitation method adopted for the synthesis of MoS_2_ doped TiO_2_ nanoparticlesis explained: 1.3 g of ammonium heptamolybdate and 0.49 g of citric acid were dissolved in 50 ml of water understirring at 90 °C for 30 min. Moreover, the pH was adjusted to 4 using ammonia. The as-prepared TiO_2_ nanoparticles (PT) and 1.27 g of thiourea was mixed in 20 ml of deionized water. The two solutions were mixed together and stirred at 90 °C for 1 h. Finally, the precipitated powder was centrifuged and washed with water/ethanol which was further annealed at 160 °C for 3 h. The sample prepared using citric acid was labelled as MoS_2_ doped TiO_2_ (MT). For comparison, 0.49 g of L-Cysteine was used instead of citric acid in the same synthesis procedure as mentioned above was labelled as L-Cysteine MoS_2_ doped TiO_2_ (L-MT). The details of the characterization tools are provided in the supplementary details.

### Photocatalytic experiment

The photocatalytic decomposition of highly carcinogenic pollutant like Congo red was examined with the irradiation of visible light (λ = 400 nm) via L-MT. In the experimental procedure, 60 mg of the synthesized photocatalyst was dispersed in 100 mL of Congo red dye solution (CR) (15 mg/L) on irradiation of visible light at various pH medium. Initially, the reaction mixture was stirred in dark without the visible light using magnetic stirrer in order to attain adsorption–desorption between the Congo red dye solution and the catalyst. From the basic mixture solution, 3 mL of reacted solution was taken for each 25 min, centrifuged and filtered for further analysis. The aliquot from the reaction was used to analyze the strength of Congo Red dye solution with the help of UV spectrophotometer (Shimadzu UV mini-1240, 200–800 nm). The effectiveness of degradation rate was derived from the following equation: Photodegradation efficiency = 1–[C/Co], where C and Co are the initial and final absorption intensity of dye solution, respectively. At the end of the photodegradation process, the used catalyst was removed, washed with distilled water and dried in oven at 120 °C to understand the reusability of the sample.

## Supplementary information

Supplementary Information.
