# Supplementary material for: Facile synthesis and defect optimization of 2D-layered MoS2 on TiO2 heterostructure for industrial effluent, wastewater treatments
Source: Sci Rep. 2020 Dec 10;10:21625. doi: 10.1038/s41598-020-78268-4 (PMC7728806; doi:10.1038/s41598-020-78268-4)
Supplement: Supplementary file 1 — Supplementary Information. [file 41598_2020_78268_MOESM1_ESM.docx]

**Facile Synthesis and Defect Optimization of 2D-layered MoS_2_ on TiO_2_ Heterostructure for Industrial Effluent, Wastewater Treatments**

Ramalingam Gopal^1*^, Maria Magdalane Chinnapan^2^, Arjun Kumar Bojarajan^1^, Naresh Kumar Rotte ^2^, Joice Sophia Ponraj^3,4^, Ravi Ganesan^5^, Ivanov Atanas ^6^, Manivannan Nadarajah ^7^, Rajesh Kumar Manavalan^8^, Joao Gaspar^4^

^1^Quantum Materials Research Lab (QMRL), Department of Nanoscience and Technology, Alagappa University, Karaikudi - 630003, Tamil Nadu, India.

^2^Department of Chemistry, St. Xavier College (Autonomus), Tirunelveli 627002, Tamil Nadu, India.

^3^Centre for Advanced Materials, Integrated-Inter-Department of LiWET Communications, Aaivalayam - Dynamic Integrated Research Academy and Corporations (A-DIRAC), Coimbatore 641046, India

^4^Department of Micro and Nanofabrication, INL–International Iberian Nanotechnology Laboratory, 4715-330 Braga, Portugal

^5^Department of Physics, Alagappa University, Karaikudi 630003, Tamil Nadu, India.

^6^Department of Mechanical, Aerospace and Civil Engineering (MACE), Brunel University, Uxbridge, UK

^7^Department of Design, Brunel University, Uxbridge, UK

^8^Institute of Natural Science and Mathematics, Ural Federal University, 620002 Yekaterinburg, Russia

^*^Corresponding authors: Dr.G.Ramalingam, Alagappa University, Karaikudi-India.

Email:ramanloyola@gmail.com

C.Maria Magdalane, Department of Chemistry, St.Xavier College (Autonomus), Tirunelveli 627002, Tamilnadu, India. Email:cmagdalane@yahoo.com

**Supplementary Information**

**Characterizations:**

X-ray diffraction patterns were recorded using Bruker advance diffractometer with a scanning rate of 5° per min with Cuk_α_ radiation source (λ=1.54060 Å) operating at 40 kV. The optical properties were analyzed using Shimadzu UV-2550 spectrophotometer in the range of 200 to 800 nm. Emission spectra with excitation wavelength of 320 nm were performed by the ultraviolet-visible-near infrared spectrophotometer (Varian Cary Eclipse Photo Luminescence, Oxford Low temperature LN2 77K set up). PHI - VERSAPROBE III – X-ray Photoelectron Spectroscopy was used with Monochromatic X-ray Beam (15 µm). Transmission electron microscopy measurements (TEM) were conducted through JEOL 2100 instrument with an operating voltage of 25KeV. Elemental analysis was performed on energy-dispersive X-ray spectrometer included in the high resolution-TEM. Electrochemical impedance spectroscopy was carried out using BioLogic SP-300 electrochemical workstation operated at 10^2^ to 10^6^ Hz frequency range with 0.5 M KCl solution containing 5.0 mM K_3_[Fe(CN)_6_]/K_4_ [Fe(CN)_6_] under open circuit potential conditions.


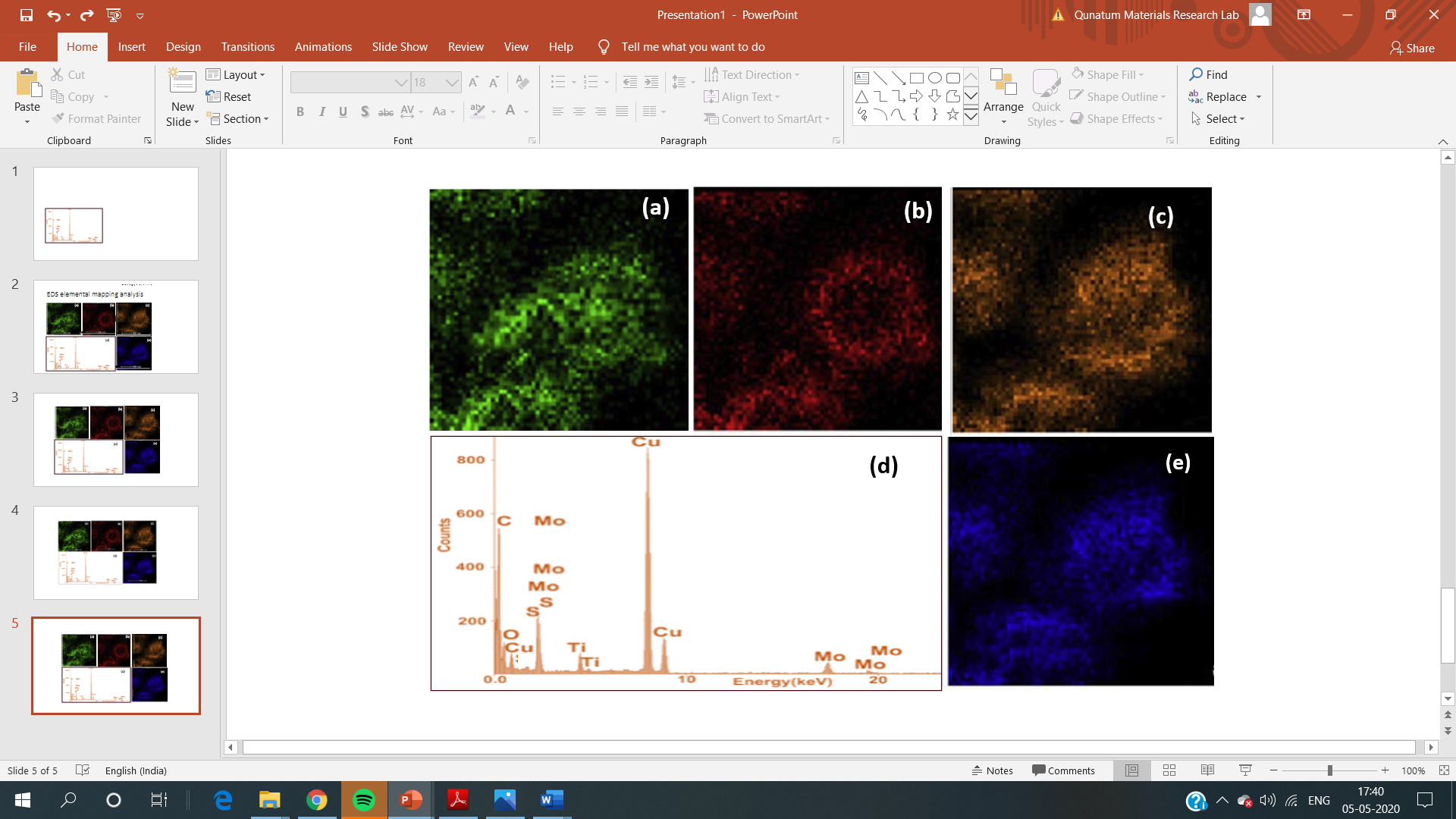


**Figure S1. Elemental spectrum (EDAX) and mapping images of Ti, O, Mo, S and figure namely identified (a), (b), (c) and (e) respectively.**

**Table. ST1.Obtained data from equivalent circuit [R_s_(Q_dl_(R_CT_(Q _dl_ (R_E_W))))]for PT, MT, L-MT**

| **Code** | **Rs /Ω cm^2^** | **Q_dl_/ F cm^-2^** | **n** | **R_CT_ /Ω cm^2^** | **Q_dl_/ F cm^-2^** | **n** | **R_E_ /Ω cm^2^** | **W/cm s^-1^** |
| --- | --- | --- | --- | --- | --- | --- | --- | --- |
| **L-MT** | 34.06 | 1.05x10^-8^ | 0.9039 | 336.7 | 2.4x10^-5^ | 0.8732 | 0.3509 | 1x10^-20^ |
| **MT** | 46.57 | 3.96x10^-9^ | 0.9582 | 426.8 | 4.211x10^-5^ | 0.8217 | 1.25x10^11^ | 1x10^-20^ |
| **PT** | 373 | 1.353x10^-5^ | 0.8635 | 488.2 | 0.001714 | 0.56 | 5320 | 0.02407 |

**Table ST2. Evaluation of photocatalysis and comparison with reported ones in dye degradation.**

| Nanocomposite | Dye | Deg.Time Deg.( %) | Light source | (mg)Cat / (mg/L)Dye, mlDye | Ref |
| --- | --- | --- | --- | --- | --- |
| 3DMoS_2_@TiO_2_ @poly (methyl methacrylate) | Methyl Orange (MO) | 50 min  95.7% | UV Light | 100mg/10(mg/L), 100 ml | ^25^ |
| MoS_2_ titania nanoheterojunctions | Methylene Blue(MB) | 100 min  90% | 20 W  tungsten halogen lamp | 10mg/10(mg/L), 100ml | ^30^ |
| TiO_2_/MoS_2_  composite | MO | 10 min  97% | 500 W xenon long-arc lamp | 400mg/(20mg/L), 100ml | ^57^ |
| 3D MoS_2_ nanosheet/TiO_2_ nanofiber heterostructures | Rhodamine B(Rh B), MO | 120 min  98.2%, 97.0% | 50 W  high-pressure  mercury lamp | 10mg/10(mg/L), 100ml | ^58^ |
| CdS–TiO_2_–Au | MO | 300 min 98% | 400 W metal halogen lamp | 150 mg/10(mg/L), 100 ml | ^59^ |
| 1D CdS NWs@TiO_2_ NPs | Mixture of MO Rh B MB | 120 sec 120 sec 180 sec  98.2% 99.2% ~100% | Sunlight | 15 mg/10(mg/L), 10 ml  15 mg/10(mg/L) , 10 ml  15 mg/10(mg/L) , 10 ml | ^60^ |
| CdS/TiO_2_ heteronanofibers | MB | 170 min 86% | 800 W xenon lamp | 100 mg/20(mg/L), 200 ml | ^61^ |
| Co/Ti LDH | CR | 75 min 99.7% | 300 W tungsten lamp | 15 mg/5(mg/L), 200 ml | ^62^ |
| ZrO_2_/TiO_2_ | RhB | 50min  100% | UV light (18 W, l = 254 nm) | 30 mg/10(mg/L), 30 ml | ^63^ |
| SnO_2_/TiO_2_ | Malachite green (MG) | 75 min, 94%. | 400 W metal halogen lamp | 60 mg/30(mg/L), 100 ml | ^54^ |
| L-cysteine capped MoS_2_@TiO_2_  (L-MT) | CR | 120 min, 97%. | 400 W metal halogen lamp | 60 mg/15(mg/L), 100 ml | Present work |
